# Evaluation of long-term effectiveness of the use of carglumic acid in patients with propionic acidemia (PA) or methylmalonic acidemia (MMA): study protocol for a randomized controlled trial

**DOI:** 10.1186/s12887-019-1571-y

**Published:** 2019-06-13

**Authors:** Marwan Nashabat, Abdulrahman Obaid, Fuad Al Mutairi, Mohammed Saleh, Mohammed Elamin, Hind Ahmed, Faroug Ababneh, Wafaa Eyaid, Abdulrahman Alswaid, Lina Alohali, Eissa Faqeih, Majed Aljeraisy, Mohamed A. Hussein, Ali Alasmari, Majid Alfadhel

**Affiliations:** 1 Genetics Division, Department of Pediatrics, King Abdullah International Medical Research Centre, King Saud bin Abdulaziz University for Health Science, King Abdulaziz Medical City, Ministry of National Guard-Health Affairs (NGHA), PO Box 22490 11426, Riyadh, Saudi Arabia; 2 Medical Genetic Section, King Fahad Medical City, Children’s Hospital, Riyadh, Saudi Arabia; 3 King Abdullah International Medical Research Centre, King Saud bin Abdulaziz University for Health Science, College of Pharmacy, King Abdulaziz Medical City, Ministry of National Guard-Health Affairs, Riyadh, Saudi Arabia; 4 Department Biostatistics and Bioinformatics, King Abdullah International Medical Research Centre, King Saud bin Abdulaziz University for Health Science, Ministry of National Guard-Health Affairs, Riyadh, Saudi Arabia

**Keywords:** Carbaglu®, Carglumic acid, Hyperammonemia, Methylmalonic acidemia, Propionic acidemia

## Abstract

**Introduction:**

Propionic acidemia (PA) and methylmalonic acidemia (MMA) are rare autosomal recessive inborn errors of metabolism characterized by hyperammonemia due to N-acetylglutamate synthase (NAGS) dysfunction. Carglumic acid (Carbaglu®; Orphan Europe Ltd.) is approved by the US Food and Drug Administration (USFDA) for the treatment of hyperammonemia due hepatic NAGS deficiency. Here we report the rationale and design of a phase IIIb trial that is aimed at determining the long-term efficacy and safety of carglumic acid in the management of PA and MMA.

**Methods:**

This prospective, multicenter, open-label, randomized, parallel group phase IIIb study will be conducted in Saudi Arabia. Patients with PA or MMA (≤15 years of age) will be randomized 1:1 to receive twice daily carglumic acid (50 mg/kg/day) plus standard therapy (protein-restricted diet, L-carnitine, and metronidazole) or standard therapy alone for a 2-year treatment period. The primary efficacy outcome is the number of emergency room visits due to hyperammonemia. Safety will be assessed throughout the study and during the 1 month follow-up period after the study.

**Discussion:**

Current guidelines recommend conservative medical treatment as the main strategy for the management of PA and MMA. Although retrospective studies have suggested that long-term carglumic acid may be beneficial in the management of PA and MMA, current literature lacks evidence for this indication. This clinical trial will determine the long-term safety and efficacy of carglumic acid in the management of PA and MMA.

**Trial registration:**

King Abdullah International Medical Research Center (KAIMRC): (RC13/116) 09/1/2014.

Saudi Food and Drug Authority (SFDA) (33066) 08/14/2014.

ClinicalTrials.gov (identifier: NCT02426775) 04/22/2015.

## Background

Propionic acidemia (PA; #606054 in the Online Mendelian Inheritance in Man [OMIM] database) and methylmalonic acidemia (MMA; OMIM #251000) are autosomal recessive inherited inborn errors of metabolism. These organic acidemias (OA) are characterized by recurrent episodes of hyperammonemic encephalopathy, which may be partially responsible for the cognitive delay seen in the majority of affected patients [1, 2]. Death can occur quickly in this population mainly owing to secondary hyperammonemia, infections, cardiomyopathy or basal ganglia stroke [3]. From an epidemiological standpoint, both OAs are very rare diseases. However, PA appears to be more common in Saudi Arabia (estimated frequency of about 1 in 3000) than in other parts of the world (1 in 100,000) [4].

The main mechanism of hyperammonemia in PA and MMA is related to the effect of acyl CoA esters on the urea cycle. Briefly, acyl CoA that has accumulated due to dysfunction of propionyl CoA carboxylase/methylmalonyl-CoA mutase inhibits the activity of N-acetylglutamate synthase (NAGS) resulting in a decrease in the production of N-acetylglutamate (NAG), a product of this enzyme. NAG is an activator of carbamoyl phosphate synthetase (CPS), and the lack of NAG results in decreased CPS activity causing hyperammonemia [5]. High concentrations of ammonia may partially saturate the “enzymatic detoxifier” of the astrocytes, impeding the brain’s capacity for self-protection and contributing to neurological dysfunction. Therefore, high levels of ammonia are a real emergency and should be treated promptly [6].

N-carbamylglutamate or carglumic acid (Carbaglu®; Orphan Europe Ltd.) is a synthetic analog of NAG that activates carbamoyl-phosphate synthetase I (CPS-I), an enzyme involved in the first and rate-limiting step of the urea cycle [7]. It is approved by the US Food and Drug Administration (USFDA) for acute and chronic treatment of hyperammonemia due to the deficiency of hepatic NAGS [8]. However, there is no current evidence supporting the use of carglumic acid for chronic management of PA and MMA. Here, we report the design and rationale of a randomized, multicenter, prospective phase IIIb study which aims primarily to compare the long-term efficacy and safety of carglumic acid (50 mg/kg/day) plus standard therapy with standard therapy alone, in decreasing the number of emergency room (ER) visits due to hyperammonemia in patients with PA and MMA.

## Methods

### Study aim, design and setting

The main aim of the study is to evaluate whether the long-term use of carglumic acid can reduce the frequency of metabolic decompensations and ER visits of PA and MMA patients due to hyperammonemia. This prospective, multicenter, open-label, randomized, parallel group phase IIIb study will be conducted at two centers in Riyadh, Saudi Arabia, (King Abdulaziz Medical City and King Fahad Medical City) and may be extended regionally and/or internationally. The study will be conducted over a 2-year period followed by a 1-month follow-up period.

Since PA and MMA are rare disorders, a multicenter design has been chosen for the study in order to allow for better recruitment of eligible patients and the subsequent generalization of the study findings. Although a crossover design would have been appropriate for a rare and chronic disease such as PA and MMA, a parallel arm design was chosen as the investigators decided that the benefits of a crossover study will be limited owing to the varying nature of PA and MMA. The use of a crossover design may lead to small, less representative and less homogenous patient groups, which might affect the interpretation of results. Furthermore, the study duration for a crossover design would have been 4 years (versus 2 years for the present parallel group design), which in turn would increase the risk of patient loss to follow-up, thereby further reducing the benefit of a crossover design. And most importantly, the parents of the patients might refuse to switch their kids to standard care arm if they have observed marked improvement due to treatment. This issue might compromise the trial midway and lead to unsuccessful outcome. Similarly, although a placebo-controlled study would have been ideal, the emergency management of acute crises using carglumic acid as a rescue medication would be difficult due to blinding.

To limit the potential for bias in this open-label trial, randomization will use a web-based system with variable block size. Variable block randomization was selected to ensure that the study groups were balanced, given the small number of patients. In addition, the endpoint criteria are objective and not influenced by investigator’s or patient’s knowledge.

### Patients

The study population will be children ≤15 years of age, since adult patients with PA and MMA generally have mild enzyme deficiency and better treatment outcomes that may not significantly change the number of ER admissions, and thus not demonstrate any potential differences between the two treatment groups.

The main study inclusion criteria are male or female patients aged ≤15 years whose parents/legal guardian agree to their participation and sign the institutional review board (IRB) approved informed consent form; patients not participating in any other trial; PA confirmed by the measurement of acylcarnitine profile, urine organic acid, propionyl CoA carboxylase in leukocytes or cultured fibroblasts or by DNA molecular testing of the PCCA or PCCB gene; MMA confirmed by the measurement of acylcarnitine profile, urine organic acid, methymalonyl CoA mutase in cultured fibroblasts or DNA molecular testing of the MUT gene*;* an expected survival of ≥6 months, defined as patients not admitted to the pediatric intensive care unit (PICU) > 2 times/year due to hyperammonemia, or asymptomatic patients diagnosed by newborn screening program or stable chronic patients who are followed up at the outpatient clinic. Genotyping will be done for all the participants to confirm the diagnosis.

The main exclusion criteria include patients with other OAs or with hyperammonemia due to other causes, receiving other investigational therapy for PA or MMA, and PA or MMA plus other inherited genetic conditions or congenital anomalies.

### Randomization and treatment

All patients who meet the study inclusion criteria and whose parents/guardians provide a written informed consent will be included in the study. Since all patients will need to receive the minimum standard of care for obvious ethical reasons, randomization during this study will compare standard therapy for PA or MMA plus carglumic acid versus standard therapy alone. Standard therapy is defined as protein-restricted diet, L-carnitine (150 mg/kg/day divided every 8 h), metronidazole (15 mg/kg/day divided every 8 h for 1 week each month) [9]. The principal investigator will conduct a protocol training session in other participating centers to ensure that the co-investigators follow the same plan. Additionally, a written management protocol including standard treatment, emergency management protocol, and nutritional management will be distributed for all participating centers. A detailed emergency card will be given for all the patients, to be shown during any emergency visit within or outside the participating centers. This card includes the patient’s chronic medications, the study arm and the standard emergency management of the patient.

All patients will be randomized (1:1) to receive carglumic acid (50 mg/kg/day) plus standard therapy or standard therapy alone for a period of 2 years. Carglumic acid will be administered twice daily in equally divided doses immediately before or with meals enterally via mouth, gastrostomy or a feeding tube depending on the clinical status of the patient (patients with PA and MMA may have a gastrostomy tube/feeding tube due to poor pharyngo-oesophageal coordination caused by neurological complications). Each 200 mg tablet of carglumic acid is dissolved in 4 mL of water to yield a concentration of 50 mg/mL, to be given as 1 mL/kg/day. Tablets should not be swallowed whole or crushed.

All patients will be instructed to keep the unopened bottle of carglumic acid (Carbaglu® 60 tablets) refrigerated at 2–8 °C, to store it at room temperature (≤30 °C) once opened, to tightly close the container after each use to protect the tablets from moisture, and to discard the bottle 1 month after opening. To monitor the patients’ compliance on the study medication, the parents and care givers will be asked about the compliance directly in the interview of each visit, which will be documented in the case report form. Additionally, the parents will be asked to bring all the medication containers, including the empty ones, to the pharmacy each visit to further monitor the compliance.

Patients will remain on medications that he/she has been taking prior to the study, except for other nitrogen scavenging drugs such as sodium benzoate, which must be discontinued at least 30 days before randomization and not taken throughout the study. Episodes of hyperammonemia during the study period may be treated with rescue medication such as intravenous sodium phenylacetate and/or sodium benzoate, with or without hemodialysis, at the clinician’s discretion. If carglumic acid is suspended during the management of an acute episode, it should be reintroduced as soon as the episode is managed.

The study is approved by the IRB at the two participating centers: King Abdulaziz Medical City, King Abdullah International Medical Research Center IRB: (RC13/116) and King Fahad Medical City IRB (14–165). The trial has been reviewed and approved by the Saudi Food and Drug Authority (SFDA) (33066), and is registered at ClinicalTrials.gov (identifier: NCT02426775). The study will be conducted in accordance with the laws and regulations of Saudi Arabia and in line with the ICH harmonized tripartite guidelines for Good Clinical Practice 1996 and the declaration of Helsinki.

### Study outcomes

The primary efficacy outcome is the number of ER visits due to hyperammonemia above the age dependent reference range during the study period. Secondary efficacy outcomes include time to first ER visit due to hyperammonemia after treatment initiation, plasma ammonia levels and levels of biochemical markers (acylcarnitine profile, urine organic acid, and plasma amino acid levels) during the study, and the number of hospitalization days during the 2-year study period.

Safety will be assessed by monitoring and recording all adverse events (AEs) and serious AEs observed during regular monitoring of hematology and blood chemistry, urinalysis, vital signs, and physical and neurological examinations.

### Study visits (Fig. 1)

All patients will be evaluated at the screening visit followed by baseline, and at 3, 6, 12, 18 and 24 months during the study. The baseline visit can be combined with the screening visit, or held within 30 days of screening. During each clinic visit, treating physicians will conduct a physical examination, blood tests and complete medical history of the patients in order to collect information on AEs and treatment compliance between the scheduled study visits. Parents/legal guardians of the patients will provide the required information to the treating physician during each clinic visit. The investigators will be asked to use the same laboratory throughout the study period for each individual patient.

**Fig. 1:**
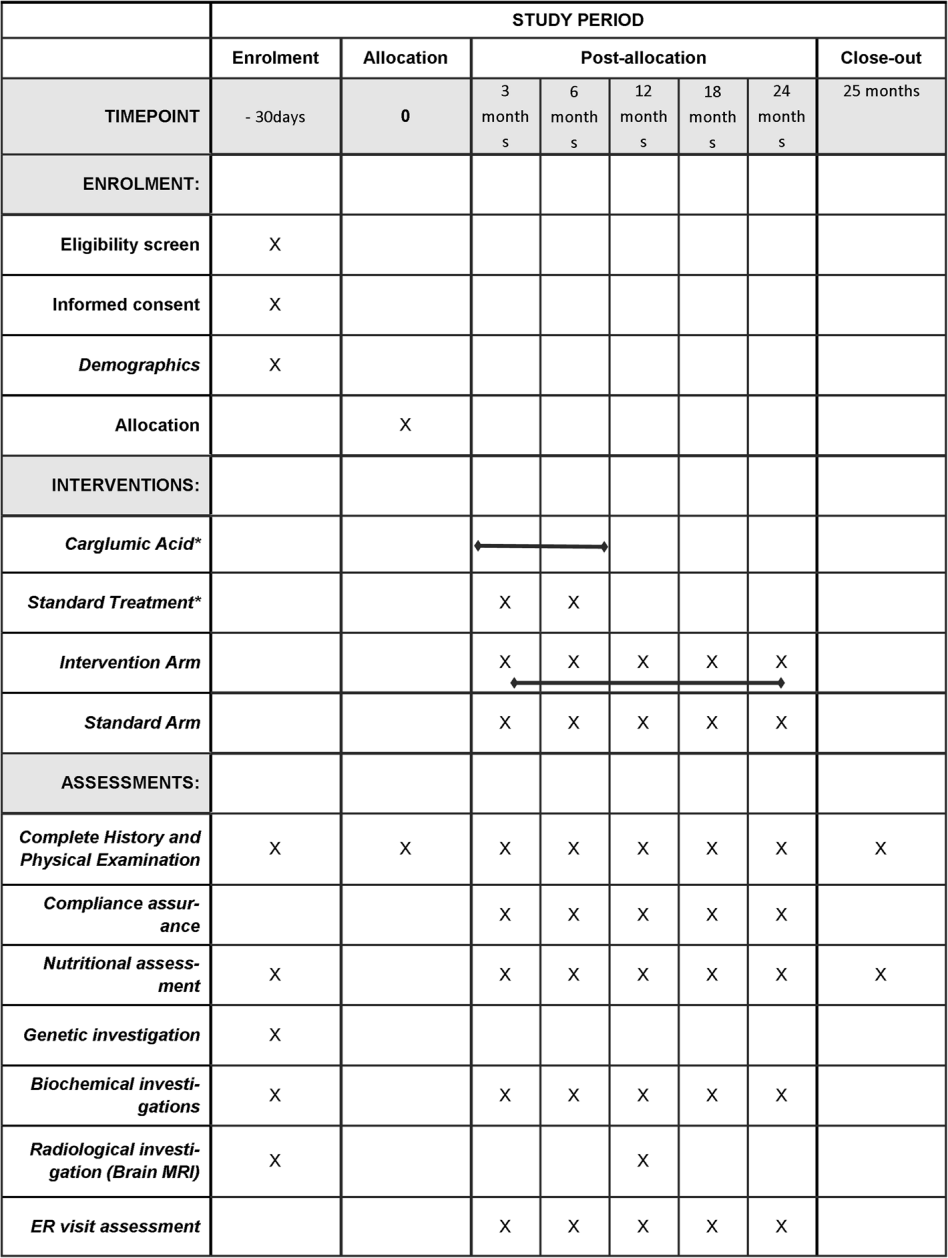
The schedule of enrolment, interventions, and assessments. *indicates only for the key symbol used for the intervention

All patients will receive a follow-up call 30 ± 7 days after the end of the study to ensure post-treatment safety. Any AEs reported during this period will be noted. In addition to the data collected at each visit, any acute decompensated episodes between scheduled visits will be recorded in a separate section in the electronic case report form (eCRF).

A specialized diet will be chosen for each study participant dependent on their age and residual enzyme activity. All patients will be required to adhere to the low protein diet and the prescribed amino acid supplements, and compliance will be assessed at each visit by a metabolic nutritionist experienced in PA and MMA who is aware of the study protocol.

### Premature discontinuation of the study

Patients will stop receiving the study drug at any time during the study if any of the following criteria are met: allergy or hypersensitivity reaction to study drug (of any grade), liver transplantation, inability to tolerate study drug (to be decided by the treating physician), acute life-threatening event related to study drug, > 30% increase in liver enzymes or long QT interval or cardiac arrhythmias, and patient or caregiver wishes to discontinue the study drug.

All patients who withdraw from the study will be included in the intention to treat analysis (ITT) based on the treatment arm they were allocated to. It will be the duty of the investigator to record the primary reason(s) and the date of the premature discontinuation of the treatment using the eCRF. Each discontinuation will be categorized as an AE (defined as any clinical or laboratory event that requires treatment discontinuation for the best interest of the patient, as diagnosed by the study investigator); withdrawal of consent (defined as patient’s desire to withdraw from further participation in the study in the absence of a medical reason to withdraw); a major protocol deviation (if the parameters recorded at each visit or the patient conduct failed to meet the protocol requirements); loss to follow-up (if the patient does not return for one or more scheduled visit(s) after treatment initiation and does not contact the investigator); other reasons such as an administrative problem, including termination of study by the sponsor.

### Study monitoring and data management

A research assistant who has expertise in data entry will enter data into a password-protected database. Data will be entered and double checked for accuracy. After resolution of any discrepancies and a combination of manual and automated data- review procedures, the final data set will be subject to a quality assurance audit.

To ensure the quality of the clinical data across all participants and sites, a clinical data management review will be performed on all subject data every 6 months. During this review, subject data will be checked for consistency, omissions and any apparent discrepancies. In addition, the data will be reviewed for adherence to protocol. To resolve any questions arising from the clinical data review process, data queries will be sent to the site for completion.

### Case report forms (CRFs) & recording of data

It is the responsibility of the Investigators to record all observations and other data pertinent to the clinical investigation. For this study an electronic CRF (eCRF) will be used.

Data on subjects during the trial will be documented in an anonymous fashion and the subject will only be identified by the subject number, and his/her initials. The Investigator must maintain source documents for each patient in the study. All information in the study database must be traceable to these source documents, which are generally maintained in the patient’s file. The source documents should contain all demographic and medical information, including laboratory data, and a copy of the signed informed consent form, which should indicate the study number and title of the trial.

### Statistical methods

#### Population

All patients included in the study will be analyzed for safety. For efficacy, all patients will be analyzed according to the treatment group to which they are randomized, using the ITT analysis. The ITT population is defined as patients who attend at least one follow-up visit during the study period. Data for patients who withdraw from the study will be included in the analysis up to the time of their withdrawal and there will be no imputation of missing data.

#### Randomization of patients

Patients will be randomized to one of the study groups using stratified variable block size randomization, where the variables for stratification will be the type of condition (PA or MMA) and the number of ER visits prior to randomization (0 visits, 1–5 visits and > 5 visits). The historical information is obtained from the medical records during the last year for those same patients that were eventually approached to be recruited into the study. Randomization will be performed using an automated randomization system that will determine the study group for a patient based on the values of the stratification variables.

#### Sample size

##### Base model (with no covariates)

Based on the historical average ER visit rate per patient per year observed in patients with PA or MMA at hospitals in Saudi Arabia, the expected baseline rate of ER visits for the standard therapy arm is expected to be approximately six events per year. Sample size calculation was based on the assumed rate ratio reduction between the two groups of 30% using Poisson regression with treatment as independent variable and total number of ER visits during the follow-up period is the dependent variable. Using the sample size calculations by Signorini [10], the study is expected to have a power of 80% for one-sided hypothesis for a sample size of 18 patients (9 for control and 9 for treatment) to detect a 30% reduction in the ER visits over the 2-year study period with 5% type I error.

##### Model with covariates

**Fig. 2:**
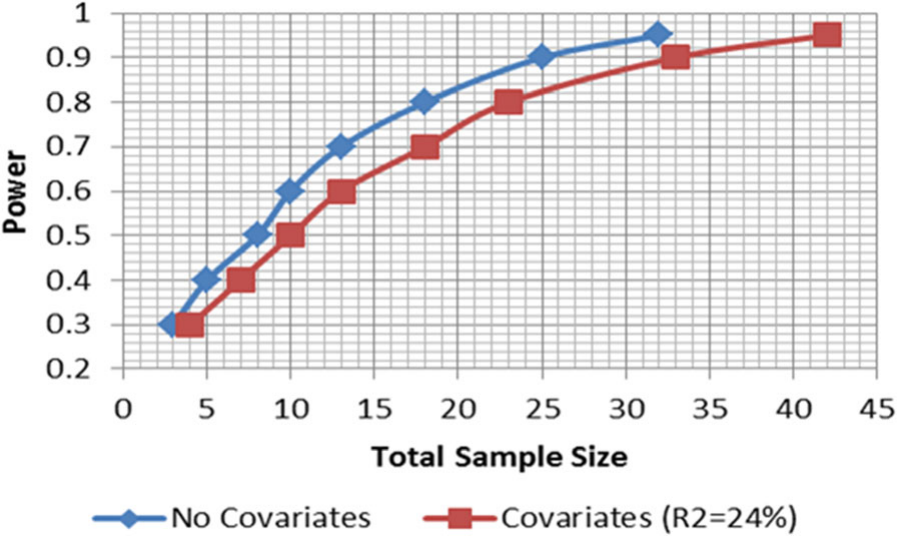
Power as a function of sample size for models with and without covariates

The maximum sample size for the study in presence of covariates was determined using the variance inflation factor (VIF) technique, which inflates the sample size proportionally to the amount of correlation between the covariates and main effect [11, 12]. The power analysis was focused on the type of the condition (PA/MMA) as it is one of the key covariates that could be less balanced between the treatment arms. Assuming that the type of condition can explain 24% of the observed variation in the study arms, the estimated total effective sample size needed for the study will be 24 patients (12 in each group). Figure 2 shows the impact of adding PA/MMA as a covariate to the model for determination of sample size. Assuming a 10% dropout rate, the expected total sample size needed for the study is 28 patients (14 patients per group).

### Interim analysis

An interim analysis will be performed after 1 year of treatment. A data and safety monitoring board (DSMB) will be convened to review the unblinded efficacy and safety data to determine whether the study can be continued for the total study duration of 2 years. Using the Haybittle-Peto stopping rules [13, 14], the study will be terminated prematurely if the treatment arm shows significant inferiority compared with the control group (*p*-value < 0.01). Inferiority will be defined as an estimated rate ratio (RR) significantly > 1.

### Ethical and regulatory standards

#### Ethics and good clinical practices

This study must be carried out in compliance with the protocol and in accordance with the laws and regulations of Saudi Arabia, and the sponsor or their representative’s standard operating procedures. These are designed to ensure adherence to Good Clinical Practice, as described in the following documents:

- ICH Harmonized Tripartite Guidelines for Good Clinical Practice 1996.
- Declaration of Helsinki, concerning medical research in humans (Recommendations Guiding Physicians in Biomedical Research Involving Human Subjects, Helsinki 1964, amended Tokyo 1975, Venice 1983, Hong Kong 1989, Somerset West 1996, Edinburgh, Scotland, October 2000, Washington 2002, Tokyo 2004, Seoul, October 2008) Brazil, October 2013.

The Investigator agrees, when signing the protocol, to adhere to the instructions and procedures described in it and thereby to adhere to the principles of Good Clinical Practice that it conforms to. A copy of the Declaration of Helsinki is provided in the Investigator study file at each site.

#### Ethics committee

Before implementing this study, the protocol, the proposed informed consent form and other information to subjects must be reviewed by an appropriate Institutional Review Board/Independent Ethics Committee (IRB/IEC). A signed and dated statement that the protocol and informed consent have been approved by the IRB/IEC must be given to the Sponsor before study commencement. The name and occupation of the chairman and the members of the IRB/IEC must be supplied to the Sponsor. Any amendments to the protocol, which need formal approval as required by local law, must be approved by this committee. The IRB may be notified of all other amendments (i.e. administrative changes).

The study may not start before written approval has been obtained for the protocol and the informed consent form.

#### Informed consent

The Investigator must explain to each subject (or legally authorized representative) the nature of the study, its purpose, the procedures involved, the expected duration, the potential risks and benefits involved and any discomfort it may entail. Each subject must be informed that participation in the study is voluntary and that he/she may withdraw from the study at any time and that withdrawal of consent will not affect his/her subsequent medical treatment or relationship with the treating physician. Any harm or adverse reactions related to the study will be treated in the study centers during and after the course of the trial.

This informed consent should be given by means of a standard written statement, written in non-technical language. The subject should read and consider the statement before signing and dating it, and should be given a copy of the signed document. No patient can enter the study before his/her informed consent has been obtained.

The informed consent form is part of the protocol, and must be submitted by the Investigator with it for IRB/IEC approval. Any changes to the proposed consent form suggested by the Investigator must be agreed to by the Sponsor before submission to the IRB/IEC and a copy of the approved version must be provided to the Sponsor after IRB/IEC approval.

#### Publication

The intention is to publish the results of the complete study at conclusion. All information obtained during the conduct of this study will be regarded as confidential and written permission from the Sponsor is required prior to disclosing any information relative to this study. A formal publication of data collected as a result of the study is planned and will be considered a joint publication by all Investigators and the appropriate Sponsor personnel. Authorship will be determined by mutual agreement.

## Discussion

Since PA and MMA are rare debilitating inborn errors of metabolism that may have life-threatening consequences, early diagnosis, and management of these OAs is of utmost importance. The implementation of newborn screening programs worldwide has helped early diagnosis of PA and MMA in asymptomatic babies in their first days of life [15]. Guidelines on acute management of hyperammonemia in the middle east region recommend the use of nitrogen scavengers, carnitine, in cases where the plasma ammonia levels are > 100 μmol/L (> 150 μmol/L in neonates), and continuous renal replacement therapy for ammonia levels > 500 μmol/L. [16] Similarly, guidelines on the diagnosis and management of PA and MMA suggest protein-restricted diet, L-carnitine, and metronidazole in the long-term management of these patients [9].

Liver transplantation may be considered in patients with PA and MMA to reduce the risk of decompensation and improve quality of life [17]. However, the decision to undertake liver transplant needs to be tailored on a case-by-case basis, for many reasons. First, the systemic nature of these mitochondrial diseases limits the expected benefits of the liver transplant, which might correct the disease partially, but not completely [18]. Second, several studies have reported long-term complications in patients after liver transplant [19–22]. Third, there are complications related to liver transplant surgery itself [21, 23]. All of these limit the use of liver transplant to severe phenotypes of PA and MMA who have a poor response to conservative medical treatment. It is recommended that liver transplant should be considered only in patients with frequent metabolic decompensations that cannot be effectively managed by medications [9]. Nonetheless, medical treatment remains the recommended first-line choice for the management of patients with PA and MMA.

Carglumic acid is currently approved as an adjunctive therapy for the treatment of acute hyperammonemia and maintenance therapy for chronic hyperammonemia in hepatic NAGS deficiency [8]. Several studies have highlighted the benefits of carglumic acid in the acute treatment of PA and MMA [24–30]. A study conducted in healthy young adults reported that carglumic acid augments ureagenesis and may be beneficial in the treatment of hyperammonemia in different clinical situations [7]. Similarly, another study in patients with PA reported that carglumic acid stimulated ureagenesis and decreased plasma ammonia levels, and may be considered as a treatment option in patients with PA [31].

However, there is limited evidence about the long-term use of carglumic acid in patients with PA and MMA. An unblinded, uncontrolled retrospective study reported that long-term treatment (median 7.9 years) with carglumic acid effectively reduced plasma ammonia levels in patients with NAGS deficiency (277 ± 359 μmol/L at baseline vs. 23 ± 7 μmol/L at 8 years). The most common AEs reported in ≥13% of patients were infections, vomiting, abdominal pain, pyrexia, tonsillitis, anemia, ear infection, diarrhea, nasopharyngitis and headache [32, 33]. Some experts in the field of inborn errors of metabolism are currently using carglumic acid for the long-term management of PA and MMA based on their experience and anecdotal evidence (unpublished data).

A retrospective study in patients with OA decompensated episodes who were treated with carglumic acid with or without ammonia-scavenging drugs or ammonia scavengers alone was conducted between January 1995 and October 2009 at 18 centers in France, Germany, Italy, the Netherlands, Spain, Turkey and the UK. A total of 57 patients with MMA, PA and isovaleric acidemia (IVA) were analyzed to determine the safety and efficacy of carglumic acid. Among the 41/57 patients included in the final analysis, 21 (51.2%) had MMA, 16 (39%) had PA and four had (9.8%) IVA. The duration of treatment for hyperammonemia with carglumic acid in the efficacy population ranged between 1 and 15 days, with an average of 5.5 days of treatment. The median time to reach plasma ammonia levels of ≤60 μmol/L after initiation of therapy was 36.5 h, which is in line with the treatment effect observed in patients with NAG deficiency. This retrospective study was used by Orphan Europe to support approval from European Medicines Agency to extend the use of carglumic acid to acute hyperammonemia due to OAs [34].

In another study, the effect of oral carglumic acid treatment (50 mg/kg/day, for 7–16 months) was investigated in patients aged 2 to 20 years with PA or MMA who were experiencing frequent episodes of metabolic decompensation and pathological levels of ammonia. The results show that in addition to short-term benefits for the acute treatment of hyperammonemia, the carglumic acid may be effective and well tolerated as a long-term treatment in patients with severe PA and MMA [35]. Furthermore, a recently published case study showed a significant decrease in plasma ammonia levels (75.7 μmol/L vs. 140.3 μmol/L before carglumic acid therapy) in a 15-year-old male patient during 6 years of treatment [36].

In conclusion, this randomized, controlled, phase IIIb trial is designed to test the hypothesis that carglumic acid (Carbaglu®) is safe and effective in the long-term management of patients with PA and MMA, as measured by the number of ER visits due to hyperammonemia.

## Data Availability

The datasets used and/or analysed during the current study are available from the corresponding author on reasonable request.

## Abbreviations

AEs: Adverse events
CPS: Carbamoyl phosphate synthetase
CPS-I: Carbamoyl-phosphate synthetase I
eCRF: Electronic case report form
ER: Emergency room
IRB: Institutional review board
ITT: Intention to treat analysis
MMA: Methylmalonic acidemia
NAG: N-acetylglutamate
NAGS: N-acetylglutamate synthase
OA: Organic acidemias
PA: Propionic acidemia
PICU: Pediatric intensive care unit
RR: Rate ratio
USFDA: US Food and Drug Administration
VIF: Variance inflation factor
